# A question of lineage

**DOI:** 10.7554/eLife.47162

**Published:** 2019-05-07

**Authors:** Sonia Sen

**Affiliations:** Tata Institute for Genetics and Society, inStemBangaloreIndia

**Keywords:** neurotransmitters, ventral nerve cord, stem cell, neuroblast, lineages, central nervous system, *D. melanogaster*

## Abstract

In the ventral nerve cord of fruit flies, neurons from the same hemilineage use the same neurotransmitter.

**Related research article** Lacin H, Chen HM, Long X, Singer RH, Lee T, Truman JW. 2019. Neurotransmitter identity is acquired in a lineage-restricted manner in the *Drosophila* CNS. *eLife* **8**:e43701. doi: 10.7554/eLife.43701

The nervous system develops from a small number of neural stems cells that generate all the different types of neurons found in an organism. First, a process of spatial patterning imparts unique molecular identities to individual neural stem cells, making them distinct from one another (Figure 1A; Holguera and Desplan, 2018). Then, a set of genes are switched on one after the other and this allows the stem cells to create different types of neurons as they divide over time (Figure 1B; Doe, 2017). More precisely, with each division, they generate one cell that will continue to be a stem cell, and one that will further divide to generate two sibling neurons (Figure 1B; Doe, 2017). A signaling pathway known as Notch is activated in one of these sibling neurons but not in the other (Truman et al., 2010).

**Figure 1.**
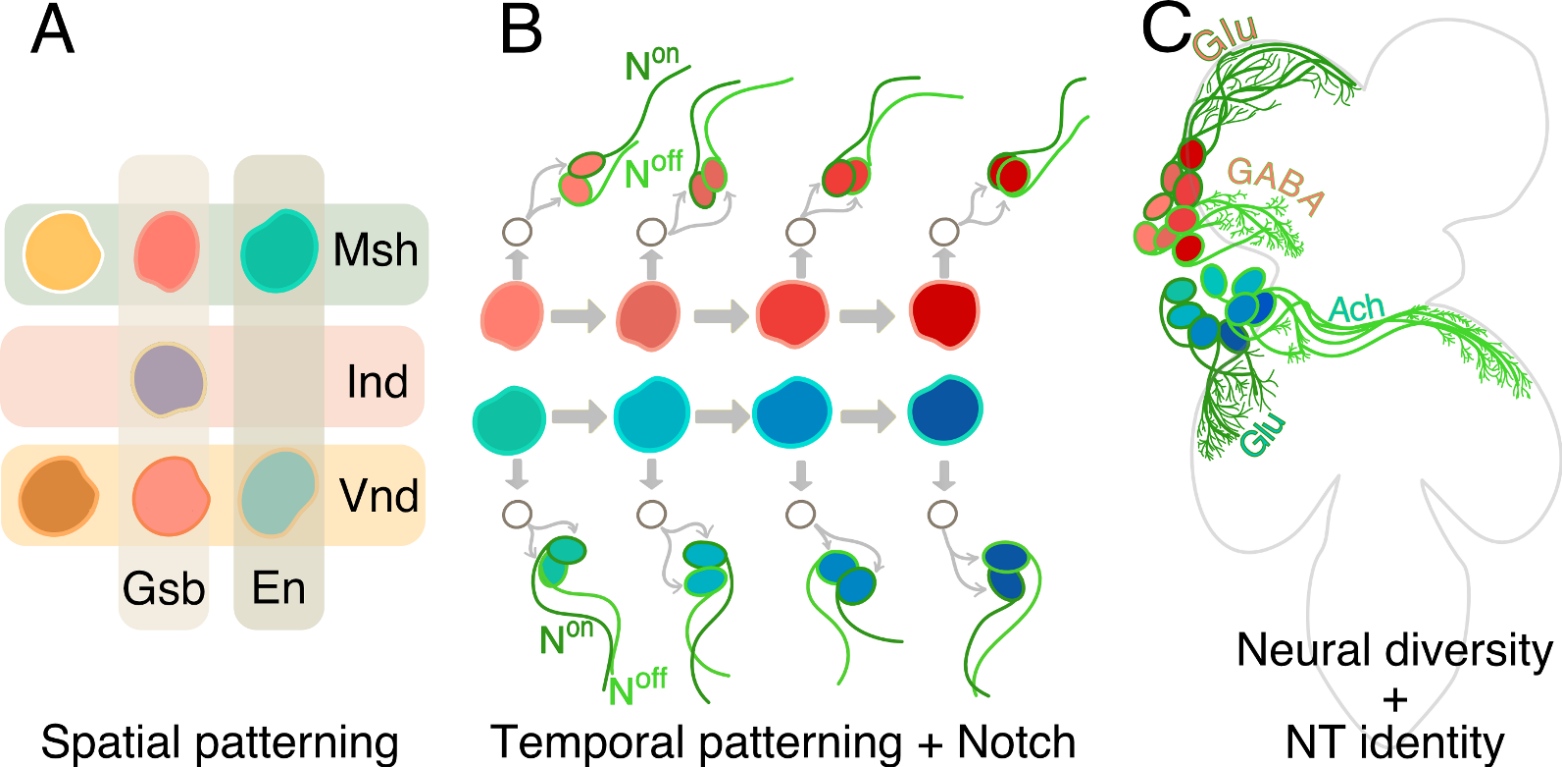
How hemilineages and neurotransmitter identity are established in the ventral nerve cord. (**A**) In the early embryo, neural stem cells form from a tissue called the neuroectoderm, where various patterning genes (Gsb, En, Msh, Ind and Vnd) are expressed. This spatial patterning process imparts unique molecular identities (different colors) to each of the stem cells. (**B**) The neural stem cells then start to divide, as shown for two neural stem cells here (red and blue-green). Each division results in a renewed neural stem cell and a ganglion mother cell (GMC; white circle). The GMC then divides to generate two neurons: one of these cells has active Notch signaling (N^on^; dark green) and the other does not (N^off^: light green). In addition, the dividing stem cells express a series of genes sequentially (represented as shades of red and blue). Throughout this temporal patterning process, each stem cell can generate progenies with slightly different identities (hence the different shades of red and blue of the daughter cells). In this way Notch signaling and temporal patterning generate diverse neurons. (**C**) As they mature, neurons coming from spatially distinct neural stem cells (red or blue) and born over time (shades of red or blue), but which share the same Notch status (light or dark green), often fasciculate together to innervate similar regions in the ventral nerve cord (four such ‘bundles’ of neurons are depicted here: red N^off^ cells; blue N^off^ cells; red N^on^ cells; blue N^on^ cells). These complex mechanisms – spatial patterning, temporal patterning and Notch status – create diverse neurons that form the developmental basis of the functional architecture of the ventral nerve cord. Now, Lacin et al. focus on three fast-acting neurotransmitter (Glutamate, or Glu; GABA; Acetycholine, or Ach) and show that all neurons within a hemilineage also share neurotransmitter identity.

Together, spatial cues, temporal information and Notch signaling generate all the diversity of neurons in the organism. Neurons born from the same neural stem cell, and which share the same ON or OFF Notch status, go on to form groups called hemilineages, which often bundle together and connect to the same areas in the nervous system (Figure 1C). These modules are therefore the developmental and functional bases of the architecture of the brain (Harris et al., 2015).

How such complex architectures then go on to execute behaviors has long fascinated neuroscientists. One approach has been to build maps of how neurons wire with each other in the brain (Schlegel et al., 2017). Combined with methods that allow access to individual neurons, these so-called ‘connectomes’ help to map the circuitry that underlies a behavior. Yet, knowing the circuit cannot explain whether a neuron activates or inhibits the cells it connects with: this depends on the neurotransmitter identity of the cell (that is, on the type of neurotransmitter it uses to communicate with other neurons). Now, in eLife, James Truman and colleagues at the Janelia Research Campus, the Albert Einstein College of Medicine, Washington University in St Louis, and the University of Washington – with Haluk Lacin as first author – report on the neurotransmitter identities of the entire ventral nerve cord of the fruit fly (Lacin et al., 2019).

The team used high quality RNA in situ hybridization to detect three fast-acting neurotransmitters – acetylcholine, glutamate and GABA – while also harnessing genetic tools and markers to label specific cells types in this region. The results show that all neurons within a hemilineage acquire the same neurotransmitter identity. Earlier studies had hinted at this: in moths and locusts, clusters of GABA neurons are generated by the same neural stem cells, and in the antennal lobe of fruit flies, related neurons share neurotransmitter identity. But by systematically analyzing the expression of the three neurotransmitters, Lacin et al. demonstrate that this may be universally true in the ventral nerve cord. That certain neurons share both connectivity and neurotransmitter identity raises interesting questions about the evolution of circuitry. Allowing circuits to develop in such a way might build redundancy and robustness into vital behaviors, while also letting complexity evolve by simply changing how many neurons are made by a stem cell.

The ways in which cells get their neurotransmitter identities seem to rely on diverse mechanisms. In the optic lobe of fruit flies, specification depends on Notch and certain transcription factors (Konstantinides et al., 2018). In the worm *C. elegans*, each glutamatergic neuron acquires its identity by using different regulatory regions of a particular gene (Serrano-Saiz et al., 2013). The mechanism in the ventral nerve cord is likely a combination of these two strategies. While neurons within a hemilineage share neurotransmitter identities, sibling hemilineages – from a single neural stem cell – can use different neurotransmitters. A simple way to explain this would be if the Notch ON hemilineage acquired one identity, while the Notch OFF hemilineage acquired another. Yet, Lacin et al. did not find a relationship between neurotransmitter identity and the Notch status of the hemilineage, nor indeed with any other lineage-specific molecular markers.

Instead, it is possible that spatial patterning, which imparts identity to the neural stem cells, predisposes the lineage towards certain neurotransmitter fates. The final identity of the neurons could then be uncovered by the Notch status of the hemilineage. Indeed, in the central brain, swapping the spatial identities of two neural stem cells also switches their neurotransmitter identities (Sen et al., 2014). Together, these data suggest that neurotransmitter identities may be acquired through complex regulatory mechanisms that are often specific to a cell: in exceptional cases, these mechanisms can even be plastic (Spitzer, 2017).

In the ventral nerve cord, a neuron only uses one of the three fast-acting neurotransmitters examined; this mutually exclusive neurotransmitter identity is also found, with some exceptions, in the optic lobes and the central brain (Konstantinides et al., 2018; Croset et al., 2018). However, the idea of ‘one neuron/one neurotransmitter’ has long been cast aside, with neurons in many organisms using several classes of neurotransmitters, including in the central brain of fruit flies (Vaaga et al., 2014; Serrano-Saiz et al., 2017; Croset et al., 2018). In these insects, fast-acting neurotransmitters are routinely co-expressed with neuropeptides and with other types of neurotransmitters such as monoamines; in addition, monoaminergic neurons often also produce neuropeptides and other types of monoamines (Croset et al., 2018; Meissner et al., 2019). So, while the three fast-acting neurotransmitter identities may be mutually exclusive in the ventral nerve cord, it is possible that they are expressed with these other types of neurotransmitters, an important avenue of research that could be pursued in the future. Meanwhile, the work by Lacin et al. gives an insight into how neurotransmitter identity might be acquired in the nerve cord, and provides an invaluable resource to understand the neural bases of behavior.
